# Tumor cell-selective apoptosis induction through targeting of K_V_10.1 via bifunctional TRAIL antibody

**DOI:** 10.1186/1476-4598-10-109

**Published:** 2011-09-07

**Authors:** Franziska Hartung, Walter Stühmer, Luis A Pardo

**Affiliations:** 1 Max-Planck-Institut für experimentelle Medizin, Hermann-Rein-Str. 3, 37075 Göttingen, Germany

**Keywords:** K_V_10.1, Eag1, scFv62-TRAIL

## Abstract

**Background:**

The search for strategies to target ion channels for therapeutic applications has become of increasing interest. Especially, the potassium channel K_V_10.1 (Ether-á-go-go) is attractive as target since this surface protein is virtually not detected in normal tissue outside the central nervous system, but is expressed in approximately 70% of tumors from different origins.

**Methods:**

We designed a single-chain antibody against an extracellular region of K_V_10.1 (scFv62) and fused it to the human soluble TRAIL. The K_V_10.1-specific scFv62 antibody -TRAIL fusion protein was expressed in CHO-K1 cells, purified by chromatography and tested for biological activity.

**Results:**

Prostate cancer cells, either positive or negative for K_V_10.1 were treated with the purified construct. After sensitization with cytotoxic drugs, scFv62-TRAIL induced apoptosis only in K_V_10.1-positive cancer cells, but not in non-tumor cells, nor in tumor cells lacking K_V_10.1 expression. In co-cultures with K_V_10.1-positive cancer cells the fusion protein also induced apoptosis in bystander K_V_10.1-negative cancer cells, while normal prostate epithelial cells were not affected when present as bystander.

**Conclusions:**

K_V_10.1 represents a novel therapeutic target for cancer. We could design a strategy that selectively kills tumor cells based on a K_V_10.1-specific antibody.

## Background

There is an intense effort invested into the search for innovative therapies that can complement classical chemotherapy, radiation and surgery to overcome the limitations derived from chemo-resistance, toxicity of non-specific drugs and incomplete elimination of tumor tissue. Resistance against conventional therapies is particularly relevant in prostate cancer [1]. Clonal selection induces the development of apoptosis-resistant, androgen-independent cells, were therapeutic prospects are relatively poor [2].

Antibodies have become essential in the diagnostic and therapeutic field and form one of the biggest classes of new drugs approved for the treatment of cancer in the last decade [3]. Single-chain antibodies (scFv) take an important role in the field, because they are less immunogenic than whole antibodies, their smaller size allows faster and deeper penetration into solid tumors, and are by definition recombinant proteins, therefore easier to produce and modify. One of such modifications rendering novel strategies for antibody-based therapies is the fusion to an effector molecule, to generate so-called bifunctional antibodies.

The tumor necrosis factor-related apoptosis-inducing ligand (TRAIL) is a promising candidate for the design of bifunctional antibodies. TRAIL is normally present as a membrane protein (memTRAIL) on immune effector cells, like natural killer cells. Binding of the trimeric TRAIL to its receptors TRAIL-R1 and TRAIL-R2 induces caspase activation and apoptosis [4], either through the extrinsic pathway alone or recruiting the intrinsic apoptotic pathway [5]. TRAIL-R1 and TRAIL-R2 have distinct crosslinking requirements for apoptosis induction [6]. TRAIL-R1 can be activated by soluble or memTRAIL, whereas TRAIL-R2 only responds to memTRAIL. TRAIL-R2 has a higher binding affinity for TRAIL, resulting in predominant binding of TRAIL to TRAIL-R2 over TRAIL-R1 [7].

TRAIL is involved in the elimination of transformed cells, e.g. cancer cells or virus-infected cells, and is effective in inhibiting tumor growth in mice [8]. Importantly, normal cells escape TRAIL-induced apoptosis for reasons currently unclear, which may involve the expression of three decoy receptors, TRAIL-R3, TRAIL-R4 and osteoprotegerin [9,10]. Some cell types are resistant to TRAIL-induced apoptosis [11], either because of a particular TRAIL receptor profile [12], through mutations affecting the mitochondrial apoptosis pathway in some type II cancer cells [13], mutations in Akt, or constitutively active NF-κB, c-FLIP or XIAP expression [14-17]. Combinational treatments with sensitizing agents are used to make cancer cells more susceptible to TRAIL-mediated apoptosis and prevent the development of resistance [18-20].

memTRAIL can undergo proteolytic cleavage and shed homotrimeric soluble TRAIL (sTRAIL). sTRAIL has a limited apoptosis induction potential (perhaps because TRAIL-R2 is less sensitive to sTRAIL than to memTRAIL [6]) and a short half-life *in vivo *[21]. The antibody-mediated binding of the scFv-TRAIL fusion proteins results in a membrane-bound TRAIL that overcomes these limitations [22-25].

Potassium channels are transmembrane proteins primarily involved in controlling the resting potential and excitability of electrically excitable cells, and in many basic cellular processes, e.g. cell cycle or proliferation [26], both in physiological and pathological conditions, including cancer. In particular the complex implication of ion channels in human prostate cancer has been repeatedly highlighted [27].

The voltage-gated potassium channel K_V_10.1 (Ether-á-go-go) shows several features that qualify it as a tumor marker. It is practically not detected in normal healthy tissue outside the CNS, but 70% of tumor cells from different origin are positive for K_V_10.1 expression [26,28-30]. Moreover, it has been shown that the inhibition of K_V_10.1 by channel blockers or down regulation of the expression leads to a decreased proliferation rate of tumor cells and impaired tumor growth *in vivo *[31,32]. *In vivo *use of K_V_10.1 inhibitors reduced tumor progression, but did not induce regression. In order to overcome this limitation, we designed a K_V_10.1-specific scFv antibody fused to sTRAIL and studied the effect in combination treatments on different prostate cancer cell lines. This approach allows taking advantage of the high tumor specificity of K_V_10.1.

## Methods

### Reagents

Polyclonal rabbit anti-TRAIL antibody (Abcam, Cambridge, UK), monoclonal mouse anti-TRAIL antibody (Sigma, Munich, Germany), horseradish peroxidase conjugated antibodies (GE Healthcare, Munich, Germany), PE-conjugated anti-TRAIL antibody, (Abcam), anti-TRAIL receptor-1 to -4 (ENZO, Lörrach, Germany), anti-activated-caspase-3 (Cell Signaling Technology, Lane, DA); cycloheximide (CHX), doxorubicin, propidium iodide, saponine, G418, roscovitine, etoposide, doxorubicin, 5-fluororuracil, cisplatin, 17-(Allylamino)-17-demethoxy-geldanamycin (17-AAG), astemizole and actinomycin D were from Sigma. RNase was from Macherey-Nagel, Düren, Germany, and Zeocin was from CAYLA-InvivoGen (Toulouse, France).

### Cell culture

Human prostate cancer cell lines DU145 (ACC261), PC3 (ACC465) and LNCaP (ACC256), HEK293 (ACC305) and CHO-K1 (ACC110) were purchased from DSMZ (Braunschweig, Germany). hTERT-RPE1 (CRL4000) and A375 (CRL-1619) were obtained from ATCC. The human prostate epithelial cell line PNT2 (ECACC95012613) was from ECACC (Salisbury, UK). Identity of prostate tumor cell lines was confirmed through expression of specific markers (DU145 AR-, ERα -, ERβ +, PSA -, DD3 -; PC3 AR-, ERα +, ERβ +, PSA -, DD3 -; LNCaP AR+, ERα-, ERβ +, PSA +, DD3 +; Prof. Paul Thelen, Department of Urology, University Hospital Göttingen). Each cell line was cultured in their respective recommended medium supplemented with 10% FCS at 37°C in humidified 5% CO_2 _atmosphere. DU145-venus cells were produced by transfecting pcDNA3-venus and selection of single clones with G418 (500 μg/ml). Transfections were done with Lipofectamine 2000 (Invitrogen, Darmstadt, Germany) as recommended by the supplier.

### Production of scFv62-TRAIL

**Figure 1:**
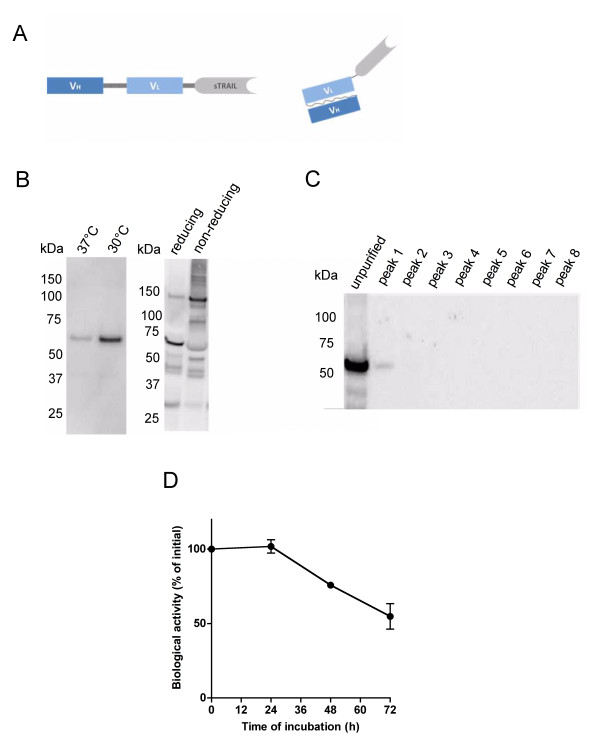
**Construction, expression and characterization of scFv62-TRAIL**. A) Schematic structure of the recombinant fusion construct scFv62-TRAIL, scFv62 (30 kDa) is genetically linked through a flexible Ser-Ser-Gly-Ser-Gly linker to soluble TRAIL (21 kDa). The monomeric scFv62-TRAIL fusion has a molecular weight of 51 kDa and the active trimer a molecular weight of ~150 kDa. B) Stable transfected CHO-K1 cells were used to produce the scFv62-TRAIL fusion protein. Cells were seeded on cell culture flasks, after allowing cell attachment normal medium was change into serum- and protein-free medium and cells were incubated at 37°C or 30°C. The scFv62-TRAIL yield in the medium was analyzed after 5 days by immunoblot using an anti-TRAIL antibody. Under non-reducing conditions scFv62-TRAIL was detected as trimers (150 kDa). C) The scFv62-TRAIL preparation was analyzed using size exclusion chromatography; the received protein peaks were analyzed by immunoblot using an anti-TRAIL antibody. D) Stability of scFv62-TRAIL in mouse serum. The scFv62-TRAIL preparation was incubated in mouse serum at 37°C up to 72 h and analyzed for its ability to induce apoptosis on DU145 cells in the presence of 5 μg/ml CHX (n = 3).

The construction of the single-chain antibody against the pore of K_V_10.1 has been described before [29]. The sTRAIL sequence was amplified from the pEGFP-TRAIL vector [33] (Addgene plasmid 10953) and cloned together with scFv62 into the multiple cloning site of pSecTag2A. The fusion protein was expressed without the tags encoded in the pSecTag2A plasmid. A pictogram of the construct is shown in Figure 1A. Transfected cells were selected with Zeocin (3 μg/ml in culture medium) and single clones were analyzed for stable secretion of scFv62-TRAIL fusion protein. For protein expression, CHO-K1 cells transfected with the pSecTag2A-scFv62TRAIL plasmid were incubated at 37°C or 30°C in Panserin C6000 (PAN Biotech, Aidenbach, Germany) (after allowing attachment for 3 h in regular medium) for five days.

Produced scFv62-TRAIL was concentrated through Centricon YM-100 (Fisher Scientific, Schwerte, Germany) and analyzed by size exclusion chromatography using a HiLoad 16/60 Superdex 200 column (Amersham Bioscience, Uppsala, Sweden). Active scFv62-TRAIL concentration was estimated by ELISA using whole monoclonal mAb62 [29] as standard.

### Caspase-3/7 assay

Caspase activity was analyzed using Caspase-Glo^® ^3/7 assay (Promega, Mannheim, Germany) according to manufacturer's instructions. Luminescence was quantified using a Victor2 plate reader (Wallac).

### Flow cytometry

For analysis of apoptosis cells were treated with scFv62-TRAIL in combination with 5 μg/ml CHX for the indicated time. Combinational treatments with the different chemotherapeutics were done with 50 U/ml scFv62-TRAIL and the indicated concentration of the particular agent for the indicated time. Induction of apoptosis was measured by flow cytometry using an Annexin V-FITC/PI staining kit (Imgenex, San Diego, CA) or Annexin V-Alexa647 (Invitrogen). Annexin V-positive cells were defined as a whole as apoptotic cells in all experiments, except for the apoptosis progression analysis where we made a distinction between early and late apoptosis.

For cell cycle analysis, cells were trypsinized, washed and resuspended in 1 ml 137 mM NaCl, 2.7 mM KCl, 100 mM Na_2_HPO_4_, 2 mM KH_2_PO_4_, 50 μg/ml propidium iodide, 0.3% saponine, 100 U/ml RNase A for 15 min at 4°C.

### Proliferation assay

Cell proliferation was measured with AlamarBlue (Biosource). The dye was added to the medium (1:10) and after 2 h incubation the fluorescence was measured in a 1420 Victor^2 ^Multilabel Counter (Ex: 544, Em: 590 nm). The relative proliferation was normalized to cell growth without inhibitor.

### Real-time PCR

Total RNA was obtained using RNAeasy (Qiagen, Hilden, Germany) and first strand cDNA was produced using SuperScript (Invitrogen). Real-time PCR was performed with 100 μg cDNA in a LightCycler 480 (Roche, Mannheim, Germany). Human transferrin receptor and actin were used as a reference. Specific mRNA content was determined using the LightCycler 480 software (Roche).

### Statistical analysis

Data were analyzed using GraphPad Prism and are represented as mean ± standard deviation (SD) between replicates. At least two independent experiments were performed for each analysis and the number of replicates (n) for each experiment is indicated. Statistical significance was evaluated by Student's t-test; *p *< 0.05 was considered as significant. *p *values are indicated by asterisks in the graphs (* p > 0.01; ** p > 0.001; ***, p < 0.001).

## Results

### Construction, expression and purification of scFv62-TRAIL

The construction of the single-chain antibody against the pore of K_V_10.1 fused to alkaline phosphatase has been described before [29]. The sequence of alkaline phosphatase was removed from the scFv62-AP construct and TRAIL was cloned from the pEGFP-TRAIL vector [33] together with a peptide linker initially into a bacterial expression plasmid (pASK-IBA2) and transformed in the *E.coli *over-expression strain BL21. After growth and induction with anhydrotetracyclin, scFv62-TRAIL was expressed and packed in inclusion bodies whose isolation requires denaturing and refolding steps. The high yields and denaturation-refolding procedure resulted in high molecular weight aggregates of the protein (data not shown) and was therefore not further pursued.

To produce scFv62-TRAIL in mammalian cells, we cloned the scFv62-TRAIL into the pSecTag2A protein expression vector, which carries the murine kappa light-chain leader peptide upstream of the multiple cloning site, and therefore directs the produced fusion protein through the ER and Golgi, resulting in excretion to the culture supernatant. Single clones were isolated from the transfected CHO-K1 cells and selected for those that showed the highest levels of secreted scFv62-TRAIL into the medium. For overexpression the cells were cultured in a protein- and serum-free CHO-K1 medium and incubated at 30°C to increase the protein yield as described by [34]. This decreased the growth rate of the CHO-K1 cells but strongly increased the scFv62-TRAIL concentration in the supernatant (Figure 1B). This method rendered amounts of the fusion construct in the active trimeric form sufficient to perform *in vitro *characterizations.

To purify active and exclude the presence of non-active monomers or high molecular weight aggregates, a size exclusion chromatography was performed. The calculated molecular weight of the scFv62-TRAIL is 51 kDa. The trimeric structure has an approximate size of 150 kDa, which could be detected on immunoblot under non-reducing conditions (Figure 1B). To exclude the presence of non-active monomers or dimers, size exclusion chromatography was performed on a with Superdex 200 column with optimal separation range from 10-600 kDa. The medium supernatant containing the scFv62-TRAIL was loaded onto the column and the different protein peaks were collected and analyzed using immunoblot and an anti-TRAIL antibody (Figure 1C). In the first peak we detected scFv62-TRAIL signal, while no TRAIL signal could be found in the later peaks containing low molecular weight proteins. The fraction containing scFv62-TRAIL was collected, concentrated and sterile filtered for further analysis.

To estimate the concentration of active scFv62-TRAIL, we performed sandwich ELISA using the recombinant fusion protein containing the epitope as antigen and detecting it by anti-TRAIL antibody. Provided that only large molecular weight complexes, compatible with trimetric TRAIL were purified, only multimeric constructs with both active antibody binding sites and TRAIL are detected. The concentration of active scFv62-TRAIL was expressed as equivalent units using the whole monoclonal antibody mAb62 as standard.

To analyze the stability of the scFv62-TRAIL fusion construct aliquots of the antibody solution were incubated in mouse serum up to 72 h at 37°C. The biological activity of the resulting material was tested on DU145 cells. After 48 h and 72 h storage in mouse serum at 37°C a reduction in the apoptosis induction potential of 25% and 45%, respectively, was observed (Figure 1D).

### K_V_10.1 expression and induction of apoptosis by scFv62-TRAIL

Prostate cancer is typically resistant against conventional therapies [1]. We chose this model because there is evidence that K_V_10.1 is expressed in human prostate cancer, and a number of cell lines with detailed characterization are available.

**Figure 2:**
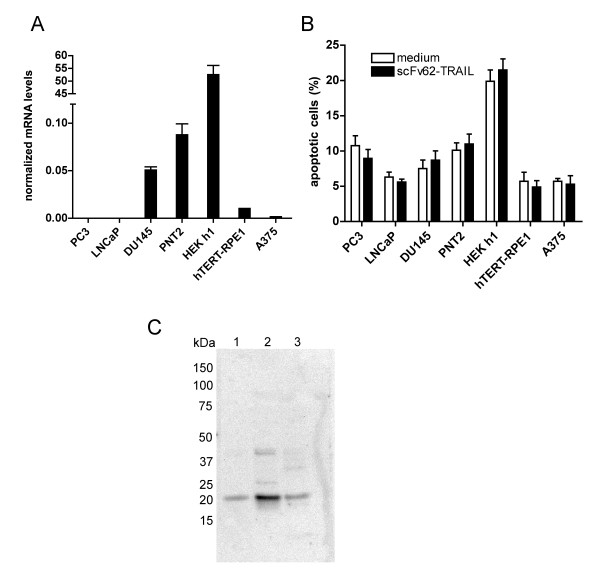
**K_V_10.1 expression analysis and apoptosis induction by scFv62-TRAIL**. A) Kv10.1 mRNA levels determined by quantitative real-time PCR. B) Apoptosis analysis using Annexin assay and flow cytometry of different cell lines treated with 50 U/ml scFv62-TRAIL or medium for 20 h (n = 2). C) Immunoblot analysis using anti-caspase-3 antibody: 1) medium supernatant of CHO-K1, scFv62 preparation, 2) scFv62-TRAIL preparation, 3) scFv62-TRAIL dialyzed through a 100 kDa cut-off membrane.

We used PNT2 (normal prostate epithelial cells immortalized by SV40 with a defective replication origin), PC3 (human prostate adenocarcinoma grade IV, androgen resistant), LNCaP (from a nodal metastasis, androgen sensitive), DU145 (from a central nervous system metastasis, androgen resistant) and A375 (melanoma). All cell lines were analyzed for expression of K_V_10.1 with real-time PCR based on the Universal Probe Library system and transferrin receptor and beta-actin as reference genes (Figure 2A). HEK293 cells transfected with K_V_10.1 (HEK h1) and hTERT-RPE1 cells were used as controls.

Among the cell lines tested, only DU145 and PNT2 showed clear K_V_10.1 expression. The A375 cells showed a weak K_V_10.1 expression. DU145 was therefore selected as tumor model for further studies. However this cell line is reported to be resistant to TRAIL-induced apoptosis due to its Bax-deficiency [35]. Therefore, scFv62-TRAIL alone was not expected to induce apoptosis in any of the cell lines mentioned above, because PNT2, hTERT-RPE1 and transfected HEK h1 are non-tumoral, LNCaP and PC3 do not express the antigen on their surface and DU145 are described to be TRAIL resistant. In the normal prostate epithelia cell line PNT2 we could also detect K_V_10.1 mRNA. Indeed, treatment of the different cell lines with scFv62-TRAIL for 18 h did not induce an increase in apoptosis levels, assessed by Annexin V-FITC and PI by flow cytometry analysis (Figure 2B). The A375 melanoma cell line has been reported to be sensitive to a TRAIL-single-chain antibody fusion construct both *in vitro *and *in vivo *[25]. This cell line was however insensitive to our K_V_10.1-specific antibody TRAIL fusion, conceivably because of low K_V_10.1 expression (see below).

We also analyzed the proapoptotic activity of scFv62-TRAIL and initially determined the induction of apoptosis by assaying caspase 3/7 activity after treating the different cancer cell lines with different doses of scFv62-TRAIL for 20 h. We observed a clear increase in caspase activity under all conditions, independently even of the presence of cells. This was due to the presence of apoptosis-independent caspase activity in the scFv62-TRAIL preparation (Figure 2C). This endogenous activity was confirmed by immunoblot and anti-caspase-3 antibody detection as a 19 kDa band. Since we could not remove this activity by extensive dialysis procedure, we exclusively performed apoptosis measurements by flow cytometry and Annexin/PI staining, which is independent of caspase-3 activity.

### Effects of scFv62-TRAIL in combination with other agents

Combination of TRAIL with other agents is common strategy to sensitize otherwise resistant cells. Cycloheximide has been often used in prostate cancer cell lines as a sensitizer, because it inhibits the cellular caspase-8 (FLICE)-like inhibitory protein (c-FLIP) and inhibitors of apoptosis (IAP) [36-38].

**Figure 3:**
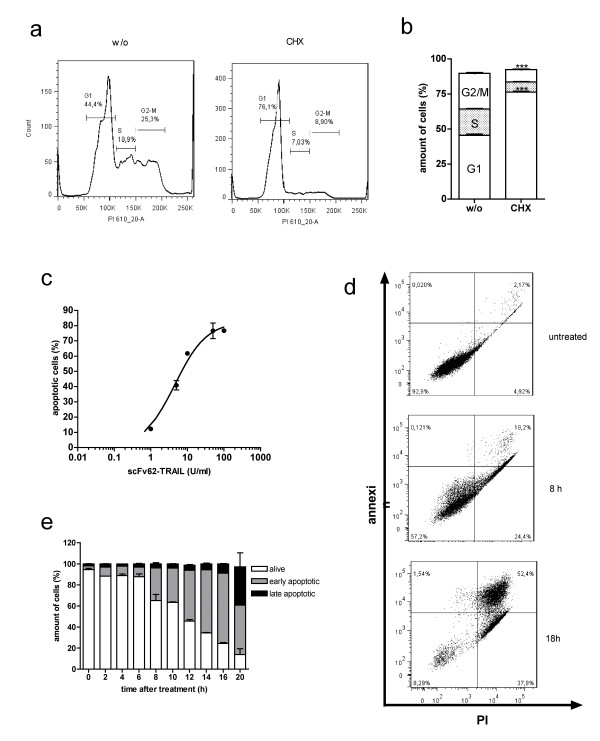
**Effect of CHX on cell cycle and in combination with scFv62-TRAIL**. A) Histograms of cell cycle analysis with flow cytometer of DU145 cells treated or non-treated with 5 μg/ml CHX and B) quantitation of cell cycle analysis. (n = 3) C) DU145 cells were treated for 18 h with different amounts of scFv62-TRAIL in presence of 5 μg/ml CHX, and subsequently analyzed for apoptosis with Annexin-FITC/PI staining in a flow cytometer (n = 3). D) and E) flow cytometer measurements and analysis: DU145 were treated with 50 U/ml scFv62-TRAIL in the presence of 5 μg/ml CHX and the progression of apoptosis was monitored at different time points with Annexin-FITC/PI staining (live cell: negative for both staining, early apoptotic: Annexin-positive, late apoptotic: Annexin and PI positive).

Treatment of DU145 cells with 5 μg/ml CHX for 24 h leads to an increase in the fraction of cells in G1 (Figure 3A and 3B), but did not affect cell viability in the tested time period. Treatment with scFv62-TRAIL in combination with 5 μg/ml CHX resulted in a massive dose-dependent apoptosis induction (Figure 3C). Over the analyzed period of 20 h, cells progressed from early apoptosis to non-viable cells. At the end of this period, 80% of the cells were apoptotic, and already one half of them showed non-competent plasma membrane (Figure 3D and 3E).

**Figure 4:**
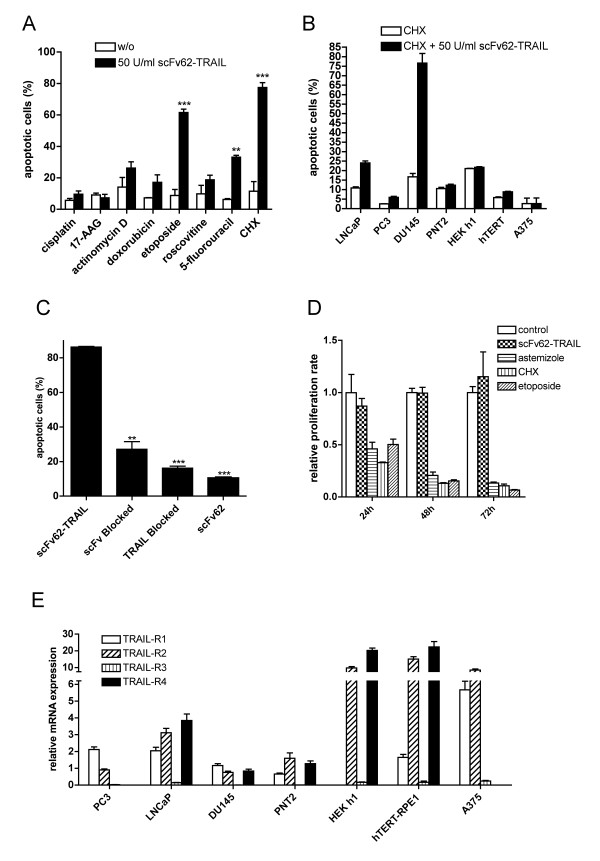
**K_V_10.1-specific apoptosis induction**. A) Annexin/PI staining and flow cytometry analysis of DU145 cells treated with scFv62-TRAIL in combination with different chemotherapeutic agents: cisplatin (10 μM), 17-AAG (5 μM), actinomycin D (800 nM), doxorubicin (1.8 μM), etoposide (50 μM), roscovitine (10 μM), 5-fluorouracil (100 μM), CHX (5 μg/ml) (n = 2). B) Cell lines were treated for 18 h with 50 U/ml scFv62-TRAIL in combination with 5 μg/ml CHX and analyzed for apoptosis with Annexin/PI staining and flow cytometry (n = 2). C) Blocking assay: DU145 were treated with scFv62-TRAIL, scFv62-TRAIL pre-incubated with anti-TRAIL antibody or scFv62-TRAIL pre-incubated with antigen, in all cases in the presence of CHX. Alternatively, cells were pre-incubated with scFv62 for 1 h and then treated with scFv62-TRAIL and CHX. As a control, DU145 cells were treated with scFv62 preparation. Apoptosis induction was analyzed with Annexin/PI staining and flow cytometry (n = 3). D) Proliferation assay: DU145 cells were treated with 50 U/ml scFv62-TRAIL, astemizole (4 μM), CHX (5 μg/ml) or etoposide (5 μM) and proliferation was measured after 24, 48 and 72 h (n = 3). E) Quantitative real-time PCR analysis of the four TRAIL receptors.

Other chemotherapeutic agents had been used to sensitize cells to TRAIL [39-41]. The construct was tested with conventionally used chemotherapeutic agents (Figure 4A). Combinational treatment with scFv62-TRAIL and etoposide or 5-fluorouracil significantly increased the apoptosis induction by scFv62-TRAIL, whereas the increase in apoptosis produced by combination with actinomycin D, doxorubicin or roscovitine did not reach statistical significance. Cisplatin and 17-AAG showed no effect. Addition of scFv62-TRAIL in combination with etoposide increased approximately 10-fold the apoptosis induction as compared with etoposide alone in DU154 cells.

Due to its low toxicity in the time window tested, CHX was used subsequently as sensitizer for further *in vitro *experiments.

### K_V_10.1- and TRAIL receptor-specific apoptosis induction

The different cell lines were treated with 50 U/ml scFv62-TRAIL in presence of 5 μg/ml CHX for 18 hours and the apoptosis induction was analyzed with Annexin/PI staining and flow cytometry (Figure 4B).

As stated before, the most sensitive cell line under these conditions was DU145. The non-cancer cell lines PNT2, HEK h1 and hTERT-RPE1 showed no apoptosis induction. In comparison to the intense apoptosis induction in DU145 cells, the K_V_10.1-negative cancer cell lines PC3 and LNCaP responded only modestly to scFv62-TRAIL treatment. The A375 cells, which have only a low expression of K_V_10.1, were not affected after combinational treatment.

To analyze the specificity of the scFv62-TRAIL and the importance of binding to the cell surface via K_V_10.1, competition experiments were performed. When the construct was pre-incubated with a fusion protein containing the epitope in order to block the antibody binding sites, the effect of scFv62-TRAIL was strongly reduced, indicating that binding to the antigen on the cell surface is required for apoptosis induction (Figure 4C). Moreover, the effect of scFv62-TRAIL was abolished when a specific anti-TRAIL antibody blocked the ligand. The single chain antibody scFv62 alone did not have any effect. Altogether, these experiments strongly indicate that both binding to K_V_10.1 on the cell surface and an active TRAIL are required to induce apoptosis.

Pre-incubation of the cells with whole anti-K_V_10.1 antibody in order to block the scFv62 recognition sites did not inhibited the effect of scFv62-TRAIL (not shown). This could be due to rapid internalization/recycling of the surface channels.

Furthermore, we analyzed the effect of scFv62-TRAIL on cell proliferation. We treated DU145 cells with scFv62-TRAIL, CHX, etoposide and the K_V_10.1-channel blocker astemizole [42] and analyzed the proliferation for 72 h (Figure 4D). CHX, etoposide and astemizole clearly reduced cell proliferation already after 24 h. But scFv62-TRAIL alone did not affect proliferation of DU145 cells.

### Analysis of TRAIL receptor expression and involvement in apoptosis induction

In order to establish if and which combination(s) of TRAIL receptors and K_V_10 are required to confer sensitivity to scFv62-TRAIL, we performed real-time PCR on the different cell lines. The data were normalized transferrin receptor and actin (Figure 4E). TRAIL-R3 was not or very weakly expressed in the different cell lines, whereas TRAIL-R4 could be detected at different expression levels in all cells, except for PC3 and A375. All cancer cell lines expressed both apoptosis-inducing TRAIL receptors, but at different ratios, with LNCaP and A375 having the highest expression rate of TRAIL-R2. Within PC3 and DU145 cells the TRAIL-R1 expression was always slightly higher than TRAIL-R2.

Among non-cancer cells, K_V_10.1 -transfected HEK h1 and hTERT-RPE1 cells showed very high TRAIL-R2 and TRAIL-R4 expression compared to the prostate cancer cell lines. The TRAIL receptor levels of PNT2 were relatively low.

**Figure 5:**
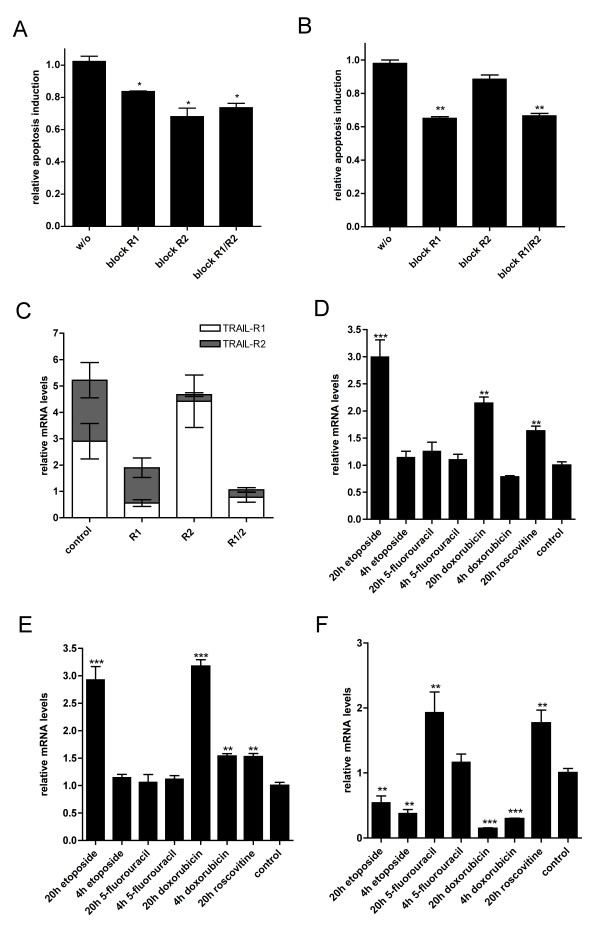
**Influence of TRAIL receptors and K_V_10.1**. A) DU145 cells were pre-incubated with anti-TRAIL-R1 antibody, anti-TRAIL-R2 antibody or a mixture of both for 2 h. Cells were then treated with scFv62-TRAIL in the presence of CHX (n = 2). B) DU145 cells were transfected with siRNA against TRAIL-R1 and/or TRAIL-R2 for 24 h and then treated with 50 U/ml scFv62-TRAIL in presence of 5 μg/ml CHX; apoptosis was measured by flow cytometry (n = 3). C) Quantitative real-time PCR analysis: DU145 cells were transfected with siRNA against TRAIL-R1 and/or TRAIL-R2 and analyzed for mRNA expression of death receptors. Quantitative real-time PCR analysis of D) TRAIL-R1, E) TRAIL-R2 and F) K_V_10.1 expression after chemotherapeutic treatment: doxorubicin (1.8 μM), etoposide (50 μM), roscovitine (10 μM), 5-fluorouracil (100 μM).

Apoptosis can be mediated via binding of TRAIL to TRAIL-R1 or TRAIL-R2. To analyze the involvement of these two receptors in apoptosis in DU145 cells we used anti-TRAIL-R1 and anti-TRAIL-R2 blocking antibodies (2 μg). After incubation of the antibodies for 1 h together with the cells we treated them with 50 U/ml scFv62-TRAIL in presence of 5 μg/ml CHX and analyzed the specific apoptosis (Figure 5A). Blocking of TRAIL-R1 reduced apoptosis induction by scFv62-TRAIL by 20%, blocking of TRAIL-R2 and both receptors resulted in a 30% apoptosis reduction. This result indicates that apoptosis induced by scFv62-TRAIL can be mediated by either receptor. However, the reduction of apoptosis was relatively modest; this can indicate incomplete blocking of the TRAIL-receptors with this approach. Therefore we decided to use siRNA to downregulate TRAIL receptors. DU145 cells were transfected with siRNA against TRAIL-R1, TRAIL-R2, or both, and subsequently treated with scFv62-TRAIL in presence of CHX (Figure 5B). Apoptosis induction was reduced by 30% after downregulation of TRAIL-R1 or both death receptors, whereas downregulation of TRAIL-R2 weakly affected the apoptotic signal. We analyzed also the influence of siRNA-mediated inhibition on the expression of other death receptors (Figure 5C). We detected an upregulation of TRAIL-R1 when TRAIL-R2 expression was downregulated and a slight reduction of TRAIL-R2 after downregulation of TRAIL-R1. This compensatory mechanism when TRAIL-R2 was downregulated caused that the total amount of messenger RNA encoding death receptors is almost the same as in the control cells, which could explain the weak reduction in the apoptosis induction.

### Chemotherapeutic treatment influences both TRAIL-R and K_V_10.1 expression

With etoposide we could sensitize DU145 cells for scFv62-TRAIL-induced apoptosis, while the other chemotherapeutic agents showed no or only a weak effect. We analyzed the influence of etoposide, 5-fluorouracil, doxorubicin and resveratrol on the expression rate of two death receptors TRAIL-R1 and TRAIL-R2. With quantitative real-time PCR an increase in TRAIL-R1 level was detected after 20 h etoposide treatment; doxorubicin showed a slight increase, whereas the other agents did not affect the expression rate (Figure 5D). The TRAIL-R2 mRNA was also only up regulated after etoposide and doxorubicin treatment for 20 h (Figure 5E).

We also tested the effect of the different chemotherapeutic agents on the expression of K_V_10.1 in DU145 cells by real-time PCR (Figure 5F). After doxorubicin and etoposide treatment for 4 or 20 h, K_V_10.1 was significantly downregulated. Treatment with 5-fluorouracil and roscovitine for 20 h increased K_V_10.1 expression, while 4 h treatment with 5-fluorouracil did not affect the K_V_10.1 expression level.

### Bystander effect of scFv62-TRAIL

**Figure 6:**
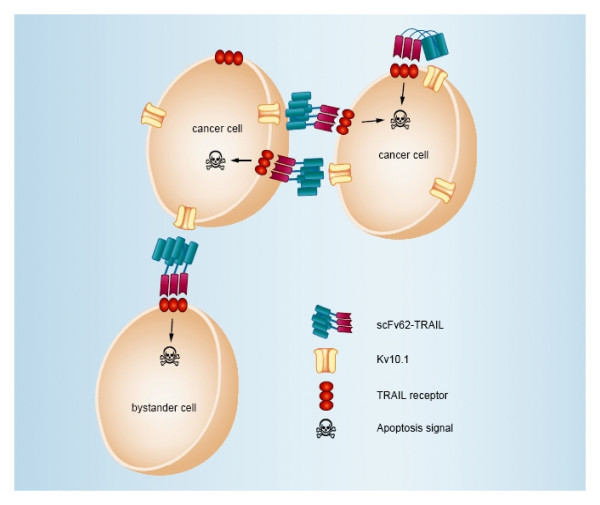
**Bystander effect**. Illustration of the scFv62-TRAIL bystander effect. Binding of scFv62-TRAIL to K_V_10.1 results in the membrane-bound form of TRAIL. Thereby, it is possible to induce apoptosis in the same cell (autocrine) or in a neighboring cell (paracrine) independently of K_V_10.1 expression. Targeting of K_V_10.1-negative cells is defined as bystander effect.

**Figure 7:**
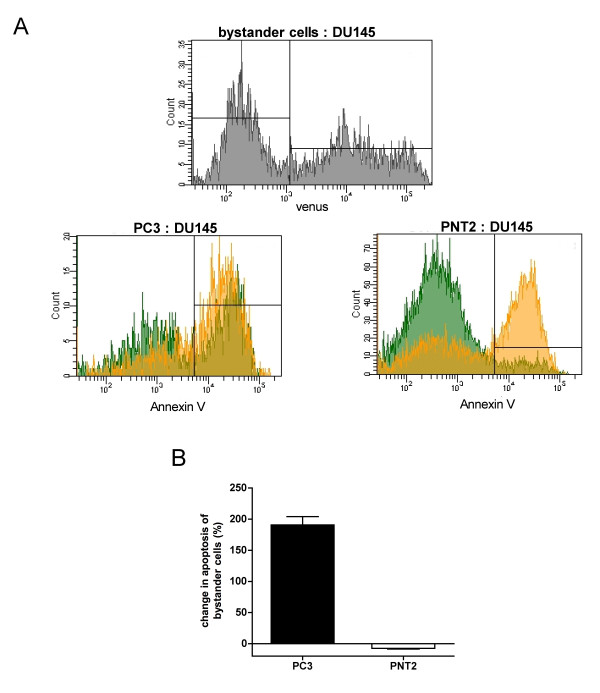
**Determination of bystander effect**. A) Upper panel. DU145 cells stably transfected with a vector expressing the fluorescent protein Venus to identify DU145 cells in mixed cultures. Two populations are clearly distinguishable by Venus fluorescence in mixed cultures. Lower panels. PC3 and PNT2 cells were co-cultured with DU145-venus cells and treated with 50 U/ml scFv62-TRAIL in the presence of 5 μg/ml CHX for 18 h. Apoptosis induction was analyzed with Annexin/PI staining and flow cytometry. Venus-positive (DU145 cells) are depicted in orange; green represents PC3/PNT2 cells (bystander cells). B) Quantification of the change in apoptosis of bystander cells was calculated.

Membrane-bound TRAIL can act through autocrine and paracrine mechanisms. Thereby, it is possible to induce apoptosis in cells not showing the antigen on their surface, provided that they are in close vicinity of positive cancer cells which provide the antigen to generate "membrane-bound TRAIL" (bystander effect; Figure 6). To study the likelihood for this to happen in our system, we co-cultured the prostate cancer cell lines PC3 (K_V_10.1-negative) and the normal prostate epithelia cells PNT2 (which expresses K_V_10.1), with the K_V_10.1-positive cancer line DU145. The DU145 cells could be identified through the stable expression of the fluorescent protein Venus (Figure 7A, upper panel).

The co-culture was treated with scFv62-TRAIL in combination with CHX. DU145 cells underwent apoptosis with similar intensity in single and co-culture. Non-cancer cells remained insensitive to scFv62-TRAIL also when co-cultured (Figure 7A and 7B). In contrast, the amount of apoptosis induction in PC3 cells was doubled when co-cultured with DU145, indicating that scFv62-TRAIL induces apoptosis in sensitive and bystander cancer cells, but leaves normal cells intact (Figure 7A and 7B).

## Discussion

Antibody-driven cancer therapy uses the high selectivity of antibodies to destroy cancer cells. Therefore, a chief factor for an efficient antibody-based cancer therapy is the targeted antigen. K_V_10.1 is a novel tumor marker with diagnostic and therapeutic potential and can be targeted by specific antibodies. We generated an antibody against the pore region of K_V_10.1 and designed a single-chain antibody genetically linked to sTRAIL to test the potential of such an approach.

The smaller yield of product achieved when using CHO-K1 cells as expression system as compared to *E.coli *is compensated by a higher quality of the product, proper folding and decreased tendency to aggregate. With a decrease of incubation temperature we increased the production of the scFv62-TRAIL [34]. The establishment and selection of monoclonal CHO-K1 producing the scFv62-TRAIL antibody renders a reliable and homogeneous system. Tags were not added on any of the fusion protein, since both the N-terminal end of the antibody part and the C-terminus of TRAIL are important for binding to the antigen or the receptor, respectively. This approach rendered scFv62-TRAIL fusion antibody in the active trimeric structure while preserving the binding capacity of the antibody.

K_V_10.1 expression was analyzed in different prostate cell lines and we confirmed the DU145 cells as K_V_10.1-positive prostate cancer cell line. Surprisingly, we detected K_V_10.1 also in normal prostate epithelia cells. The K_V_10.1 expression of these cells is likely an effect of the SV40 virus immortalization.

We initially used a 96-well format assay for active caspase 3/7. This approach turned out to be unsuccessful because of the non-specific presence of caspase-3, apparently integrating high-molecular-weight complexes. It is conceivable that during production of proteins, many CHO-K1 cells undergo normal apoptosis and apoptotic proteins of the lysed cells are released into the medium. Caspase-3 production is not related to TRAIL in the scFv62-TRAIL expression, because it is also detected in the scFv62 (single chain without TRAIL) preparations. Further apoptosis analyses were performed using Annexin/PI staining and flow cytometry, an active-caspase-3 independent method. It is unlikely that the presence of caspase-3 in the supernatants is responsible for the induction of apoptosis, since the scFv62 preparation did not induce apoptosis, although it contains also caspase-3.

TRAIL selectively kills a variety of tumor cell lines while sparing the majority of normal cells from apoptosis. The TRAIL apoptosis pathway acts independently of p53, which makes it a potentially effective weapon against chemo- or radio-resistant tumors [43]. Cytotoxicity and enhanced survival or even proliferation of resistant tumor cells hampered the clinical use of sTRAIL. Combination treatments are used to overcome the resistance and sensitize resistant tumor cells for TRAIL-induced apoptosis. Nevertheless, the short half-life and rapid blood clearance are drawbacks of sTRAIL *in vivo *[8]. Our scFv62-TRAIL antibody showed a half-life of ~72 h in mouse serum at 37°C, sufficient for *in vivo *use. The reported toxicity of TRAIL to normal prostate epithelial cells seems to be a problem of high-molecular-weight aggregates deriving form bacterial expression systems [44] and should not be a concern with our preparation. Using CHO-K1 cells we were able to express correctly folded and non-aggregated scFv62-TRAIL fusion proteins.

Different prostate cancer cell lines have been characterized regarding their susceptibility to TRAIL. We selected DU145 cells because of the high K_V_10.1 expression level and their known resistance to TRAIL-induced apoptosis. As control cells we used the K_V_10.1-negative cell lines PC3 and LNCaP as well as the normal epithelial cell line PNT2. All tested cell lines are relative resistant against low doses (50 U/ml) of the scFv62-TRAIL fusion construct as single agent, as previously reported for other antibody-TRAIL constructs [45,46]. Resistance of cancer cells is mediated by multiple defects in the TRAIL signaling pathway, e.g. downregulation of death receptors, mutations in the mitochondrial pathway or overexpression of anti-apoptotic proteins, like c-FLIP or XIAP [12,13,15,16]. Several studies highlight the requirement of sensitizing agents for effective TRAIL-induced apoptosis and prevention against the development of resistance [46]. We treated the prostate cells with scFv62-TRAIL in combination with CHX, and detected a strong apoptosis induction within 20 h in DU145 cells, whereas the K_V_10.1-negative cancer and normal epithelial cells remained unaffected. Furthermore, the blocking experiments strongly indicated that both binding to Kv10.1 to the cell surface and an active TRAIL are required to induce apoptosis, and confirmed the specificity of scFv62-TRAIL. This observation supports our basic idea of K_V_10.1-selective targeting of cancer cells via antibody-based therapies.

We studied also the melanoma cell line A375, which expresses K_V_10.1 and has been described to be sensitive for TRAIL fused to an antibody [25]. However, we could not detect an apoptosis-inducing effect on this cell line neither using scFv62-TRAIL alone nor in combination with CHX. This could be attributed to the fact that the apoptosis-inducing effect of the antibody-TRAIL fusion construct described by Bruyn *et al. *is not only based on TRAIL, but also on the blocking of the tumorigenic MCSP (melanoma chondroitin sulfate proteoglycan) signaling mediated by the fused antibody.

We analyzed the expression levels of the four TRAIL receptors in the different cells with real-time PCR. All prostate cancer cells showed TRAIL-R2 expression, which has a higher affinity for the ligand but requires a membrane-bound form for apoptosis induction. This observation may explain the low efficacy of sTRAIL against prostate cancer cells in other studies. An up-regulation of TRAIL-R2 expression and increasing sensitivity to TRAIL during tumor progression has been reported for prostate cells [47]. Even though DU145 are androgen-independent and therefore less differentiated cancer cells than LNCaP and PC3, TRAIL-R2 expression is even lower in these cells.

TRAIL-R4 mRNA was found in DU145 and LNCaP cells, but not in PC3. As a non-apoptosis inducing receptor TRAIL-R4 stimulates the NF-κB pathway and high NF-κB levels lead to TRAIL resistance [9,48]. Using CHX as protein synthesis inhibitor we would inhibit the NF-κB-induced protein expression and restore the sensitivity to TRAIL-induced apoptosis in DU145 cells. CHX could also increase sensitivity of DU145 cells by restoring the cross talk between the extrinsic to the intrinsic pathway interrupted by the loss of function of Bax [35] or by inducing accumulation of cells in the G1 phase of the cell cycle [49]. TRAIL-R4 and TRAIL-R2 could be involved in the resistance against TRAIL-induced apoptosis in normal cells, because HEK h1 and hTERT-RPE1 show high mRNA levels of both.

Etoposide has been described to sensitize cancer cells for TRAIL-induced apoptosis by up-regulation of TRAIL-R1, TRAIL-R2, Bax and Bak [50]. We detected an increase in the TRAIL-R1 and TRAIL-R2 mRNA expression level in DU145 cells after 20 h etoposide treatment. The up-regulation of the death receptors and the descript activation of the intrinsic pathway explain the restored sensitivity to TRAIL-induced apoptosis in DU145 cells. The cause of resistance to TRAIL is a combination of diverse alterations in the TRAIL signaling of the particular tumor cell, therefore optimized combinational treatments for scFv62-TRAIL need to be determined for every cancer type in further studies.

A combinational therapy of scFv62-TRAIL with etoposide appears to be a promising option for *in vivo *application, because of the strong sensitizing effect for TRAIL in DU145 cells. Unfortunately, we observed a downregulation of K_V_10.1 after etoposide treatment. Careful analyses of K_V_10.1 protein expression will be necessary during *in vivo *long-term treatment to avoid a reduction in therapeutic efficiency as a result of antigen downregulation.

We wanted to investigate if scFv62-TRAIL mediates apoptosis via TRAIL-R1 or TRAIL-R2 by blocking the receptor with specific antibodies. It is not completely clear which death receptor (or if both receptors) are important for apoptosis induction via scFv62-TRAIL. However, the expression of TRAIL-R1 and TRAIL-R2 seems to be connected, because siRNA-mediated downregulation of TRAIL-R2 in DU145 cells dramatically increases TRAIL-R1. This can explain why we observed no decrease in apoptosis induction after down-regulating TRAIL-R2, because increased TRAIL-R1 expression can compensate the TRAIL-R2 downregulation. Furthermore, this effect also suggests an involvement of both death receptors in the scFv62-mediated apoptosis induction. The possible role of decoy receptors R3 and R4 cannot be discarded at this point. Conceivably, sensitivity is determined by the precise constellation of death and decoy receptors and not by the abundance of a particular receptor type.

Apoptosis can be induced in an autocrine manner by binding to TRAIL receptors on the same cell or in a paracrine one, with binding to receptors on a neighboring cell. Thereby also neighboring tumor cells devoid or with low expression of the target antigen can be effectively eliminated by the so-called bystander effect [51]. We could detect potent bystander effect of scFv62-TRAIL against K_V_10.1-negative cancer cell, whereas normal prostate epithelia cells are not affected. This confirms the retained tumor-selectivity of the scFv62-TRAIL antibody construct.

## Conclusions

In summary, we describe a system based on the combination of two tumor-specific features, such as K_V_10.1 expression and sensitivity to TRAIL. This renders an agent able to induce apoptosis *in vitro *in sensitized K_V_10.1-expressing prostate cancer cells and also in neighboring cancer cells without K_V_10.1 on their surface, but sparing healthy cells.

## List of abbreviations

TRAIL: TNF-Related Apoptosis-Inducing Ligand.

## Competing interests

WS and LAP are shareholders at iOnGen AG.

## Authors' contributions

FH participated in the study design, carried out the experiments, participated in the analysis and drafted the manuscript. WS participated in the design of the study and in writing the manuscript. LAP participated in the study design, data analysis and in writing the manuscript. All authors read and approved the final manuscript.
